# *Hoxa9* and *Hoxa10* induce CML myeloid blast crisis development through activation of *Myb* expression

**DOI:** 10.18632/oncotarget.22008

**Published:** 2017-10-24

**Authors:** Vijay Negi, Bandana A. Vishwakarma, Su Chu, Kevin Oakley, Yufen Han, Ravi Bhatia, Yang Du

**Affiliations:** ^1^ Department of Pediatrics, Uniformed Services University of the Health Sciences, Bethesda, MD, USA; ^2^ Division of Hematology/Oncology, Department of Medicine, University of Alabama at Birmingham, Birmingham, AL, USA

**Keywords:** Hoxa9, Hoxa10, Myb, chronic myeloid leukemia, blast crisis

## Abstract

Mechanisms underlying the progression of Chronic Myeloid Leukemia (CML) from chronic phase to myeloid blast crisis are poorly understood. Our previous studies have suggested that overexpression of *SETBP1* can drive this progression by conferring unlimited self-renewal capability to granulocyte macrophage progenitors (GMPs). Here we show that overexpression of *Hoxa9* or *Hoxa10*, both transcriptional targets of *Setbp1*, is also sufficient to induce self-renewal of primary myeloid progenitors, causing their immortalization in culture. More importantly, both are able to cooperate with *BCR/ABL* to consistently induce transformation of mouse GMPs and development of aggressive leukemias resembling CML myeloid blast crisis, suggesting that either gene can drive CML progression by promoting the self-renewal of GMPs. We further identify *Myb* as a common critical target for *Hoxa9* and *Hoxa10* in inducing self-renewal of myeloid progenitors as *Myb* knockdown significantly reduced colony-forming potential of myeloid progenitors immortalized by the expression of either gene. Interestingly, *Myb* is also capable of immortalizing primary myeloid progenitors in culture and cooperating with *BCR/ABL* to induce leukemic transformation of mouse GMPs. Significantly increased levels of *MYB* transcript also were detected in all human CML blast crisis samples examined over chronic phase samples, further suggesting the possibility that *MYB* overexpression may play a prevalent role in driving human CML myeloid blast crisis development. In summary, our results identify overexpression of *HOXA9*, *HOXA10*, and *MYB* as critical drivers of CML progression, and suggest *MYB* as a key therapeutic target for inhibiting the self-renewal of leukemia-initiating cells in CML myeloid blast crisis patients.

## INTRODUCTION

CML, primarily induced by the expression of an abnormal BCR/ABL fusion tyrosine kinase, is characterized by a gradual progression from a benign chronic phase displaying hyperproliferation of normally maturing myeloid cells to a lethal blast crisis, in many cases resembling AML where myeloid differentiation is impaired. Although treatment of chronic-phase patients with tyrosine kinase inhibitors (TKIs) has been shown to induce disease remission and to prevent progression in most cases, a significant population of patients still developed blast crisis for which treatment options are very limited. Targeting the self-renewal of leukemia-initiating cells (LICs) represents a promising strategy for the treatment of leukemia; however, the self-renewal mechanisms for LICs in CML myeloid blast crisis remain unclear. Different from the chronic phase, where the LICs likely are derived directly from HSCs [1, 2], an origin from the more differentiated granulocyte-macrophage progenitors (GMPs) has been suggested for LICs in CML myeloid blast crisis as the GMP population in this phase is significantly expanded and displays increased self-renewal capability [3]. While abnormal activation of a number of genes/pathways has been implicated in the self-renewal of GMPs in myeloid blast crisis, including WNT/β-CATENIN, *RUNX1*, *RUNX1/EVI1*, *RUNX1/PRDM16*, *GATA-2*, and *Msi2* [3–8], direct evidence in supporting this concept has come from studies on several genes including *NUP98/HOXA9*, *Hes1* and *Setbp1*, showing that they can cooperate with *BCR/ABL* to transform GMPs into LICs for development of CML myeloid blast crisis *in vivo* [2, 9, 10]. Identification and characterization of downstream targets of these genes should lead to a better understanding of the generation and self-renewal mechanisms of LICs of CML myeloid blast crisis.

Homeobox transcription factor genes *Hoxa9* and *Hoxa10* have been identified as critical activation targets of *Setbp1* for its ability to induce self-renewal of myeloid progenitors and development of CML myeloid blast crisis [10]. Both *Hoxa9* and *Hoxa10* are validated oncogenes capable of inducing AMLs [11, 12]. Past studies also have suggested that overexpression of either gene may stimulate the self-renewal of myeloid progenitors. Immortalized myeloid progenitor lines can be generated by transducing mouse bone marrow progenitors with retrovirus expressing *Hoxa9* [13]. Overexpression of either gene in GMPs also has been found to induce serial re-plating activity [14]. In addition, both are also key targets of MLL translocation genes, which have been shown to induce self-renewal and transformation of GMPs *in vivo* [14]. Therefore, it is possible that overexpression of *HOXA9* or *HOXA10* alone may be sufficient to cooperate with *BCR/ABL* to induce transformation of GMPs, leading to CML progression into myeloid blast crisis; however, this hypothesis has not been tested.

Here we show that overexpression of *Hoxa9* or *Hoxa10* is sufficient to confer unlimited self-renewal capability to myeloid progenitors *in vitro* and to cooperate with *BCR/ABL* to induce transformation of GMPs *in vivo*, leading to the development of myeloid blast crisis. Our study also identifies *Myb* as a shared critical target of *Hoxa9* and *Hoxa10* for their self-renewal inducing activity. Our findings on the capability of *Myb* to drive CML myeloid blast crisis development and the prevalent overexpression of *MYB* in CML myeloid blast crisis patients further suggest that inhibition of MYB activity could be an effective strategy for inhibiting the self-renewal of LICs in CML myeloid blast crisis.

## RESULTS

### Both *Hoxa9* and *Hoxa10* are capable of inducing immortalization of myeloid progenitors

**Figure 1:**
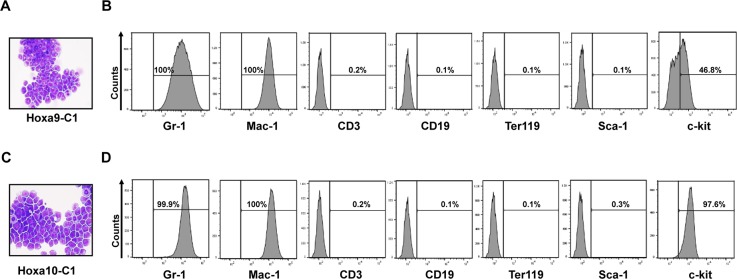
Constitutive expression of *Hoxa9* or *Hoxa10* alone is capable of inducing immortalization of myeloid progenitor cells Representative Wright-Giemsa staining and FACS analysis of indicated marker expression of cells immortalized by transduction with retroviruses expressing either Hoxa9 (**A** and **B**) or Hoxa10 (**C** and **D**) after passaging in liquid media containing SCF and IL-3 for 2 months.

We have found previously that overexpression of *Setbp1* can confer self-renewal capability to myeloid progenitors, and that this activity of *Setbp1* is critically dependent on its activation of *Hoxa9* and *Hoxa10*. To further explore the mechanism(s) underlying *Setbp1*-induced self-renewal, we tested whether overexpression of *Hoxa9* or *Hoxa10* alone also is sufficient to confer unlimited self-renewal capability to myeloid progenitors by first assessing their ability to immortalize myeloid progenitors in culture. We transduced primary murine myeloid progenitors with MSCV retroviruses expressing *Hoxa9* cDNA (*MSCV-Hoxa9-PGK-Neo*) or *Hoxa10* cDNA (*MSCV-Hoxa10-PGK-Puro*), and subsequently passaged the cells in culture in the presence of stem cell factor (SCF) and interleukin-3 (IL-3) as described previously [10]. Infections were carried out using a low viral titer of 1 × 10^5^ cfu, which was shown previously to be insufficient for empty MSCV retroviral vectors to induce immortalization through insertional mutagenesis [15]. As controls, cells infected by same titers of empty MSCV viruses (*MSCV-PGK-Neo* and *MSCV-PGK-Puro*) were passaged together. At two weeks after infection, significant reduction in proliferation and increase in macrophage differentiation were observed in the cultures of empty virus-infected cells. In contrast, cultures from *Hoxa9* or *Hoxa10* virus-transduced cells were still dominated by proliferating myeloid cells. These cells are immortalized as they can be continuously passaged for six months until the experiments are terminated (Figure 1). Consistent with their myeloid lineage, these cells express myeloid markers including Gr-1 and Mac-1, and are negative for markers of lymphoid and erythroid lineages (Figure 1). Similar to progenitors immortalized by *Setbp1*, these cells are dependent on IL-3 for their proliferation (data not shown). Immortalized myeloid progenitor lines with similar phenotypes also could be established from purified mouse GMPs and 5-FU-treated bone marrow progenitors by transduction with *Hoxa9* and *Hoxa10* viruses (data not shown). These results suggest that either *Hoxa9* or *Hoxa10* alone can induce immortalization of myeloid progenitors.

### *Hoxa9* cooperates with *BCR/ABL* to induce transformation of GMPs *in vivo*

**Figure 2:**
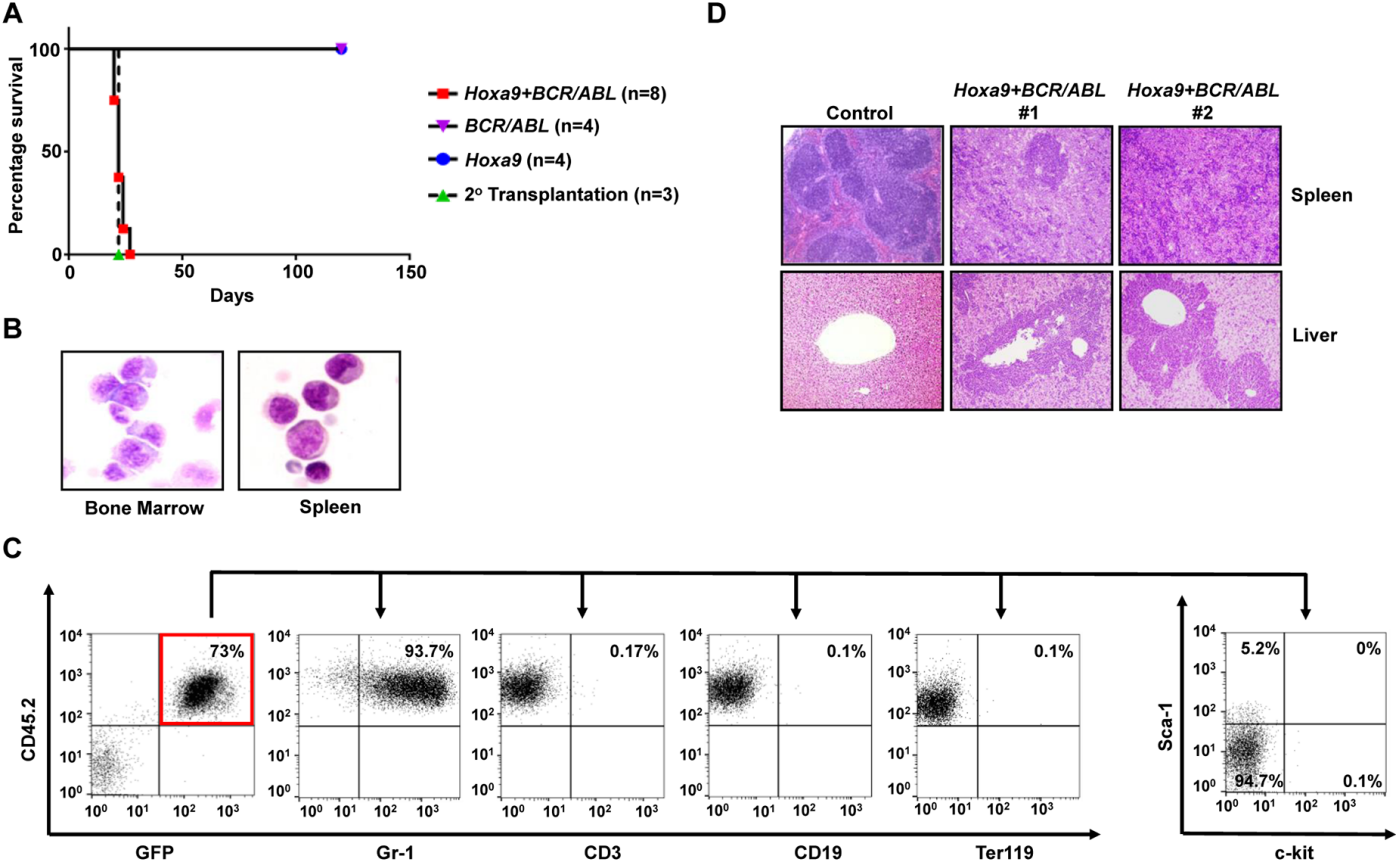
*Hoxa9* cooperates with *BCR/ABL* to induce development of CML myeloid blast crisis **(A)**. Survival curves of lethally-irradiated B6-Ly5.2 mice receiving GMPs transduced with *MSCV-Hoxa9-PGK-Neo* virus alone, *MSCV-BCR/ABL-IRES-GFP* virus alone, the combination of both viruses, or 1 × 10^6^ spleen cells from primary leukemic mice. **(B)**. Representative cytospin analysis of bone marrow and spleen cells from leukemic mice. **(C)**. Representative FACS analysis of GFP and CD45.2 double positive leukemia cells from the bone marrow of leukemic mice using the indicated antibodies. Numbers represent the percentages of gated events. **(D)**. H&E staining of spleen and liver tissue sections showing leukemic infiltration in two *Hoxa9+BCR/ABL* leukemic mice in comparison to a healthy control mouse.

The ability of *Hoxa9* and *Hoxa10* to immortalize myeloid progenitors *in vitro* suggested that they may also help confer limitless self-renewal potential to GMP-derived LICs in CML myeloid blast crisis. We first tested this hypothesis with *Hoxa9*. We co-transduced GMPs (purified from C57BL/6 mice) with MSCV retrovirus expressing *BCR/ABL* (*MSCV-BCR/ABL-IRES-GFP*) and *Hoxa9*-expressing *MSCV-Hoxa9-PGK-Neo* virus. Co-transduced GMPs were then transplanted into lethally irradiated congenic B6-Ly5.2 recipient mice along with supporting bone marrow. GMPs infected singly by the same titer of either virus were transplanted into mice as controls. Interestingly, all mice receiving co-transduced GMPs became sick within the first four weeks of transplantation with dramatically enlarged spleens (Figure 2A and data not shown). Cytospin analysis of the bone marrow and spleen cells of the moribund mice showed domination of abnormal cells resembling myeloid blasts (Figure 2B), which represent 32 ± 3.2% (Mean ± SD) of all nucleated cells in the bone marrow, suggesting the development of CML myeloid blast crisis. In support of this notion, more than 90% of these cells in the bone marrow were positive for myeloid markers Gr-1, and negative for CD3, CD19 and Ter119, which are markers for T cells, B cells, and erythroid cells, respectively (Figure 2C). Five to 11% of the cells in the bone marrow and spleen also were positive for Sca-1, while less than 1% of the cells were positive for c-kit (Figure 2C). Pathological examination of these mice further showed extensive spleen and liver infiltrations by the abnormal cells (Figure 2D). As expected, these cells expressed high levels of *BCR/ABL* and *Hoxa9* mRNAs (Supplementary Figure 1). Transplantation of 1 × 10^6^ spleen cells from these moribund mice into secondary recipient mice also led to development of same disease in four weeks (Figure 2A). We also found that neomycin-resistant colonies formed by the spleen cells are positive for GFP (data not shown), further suggesting the disease development required both *Hoxa9* and *BCR/ABL* expression within the same cells. In contrast, consistent with previous studies demonstrating the inability of *BCR/ABL* to induce self-renewal and transformation of GMPs [1, 2, 9, 10], mice transplanted with GMPs that were singly transduced with *BCR/ABL* virus remained healthy for four months (Figure 2A). Mice receiving cells transduced by *Hoxa9* virus alone also did not develop any leukemias, suggesting *Hoxa9* by itself is not sufficient to transform GMPs. In combination, these results suggest that *Hoxa9* is capable of cooperating with *BCR/ABL* to induce development of CML myeloid blast crisis.

### *Hoxa10* is also capable of cooperating with *BCR/ABL* to induce GMP transformation *in vivo*

**Figure 3:**
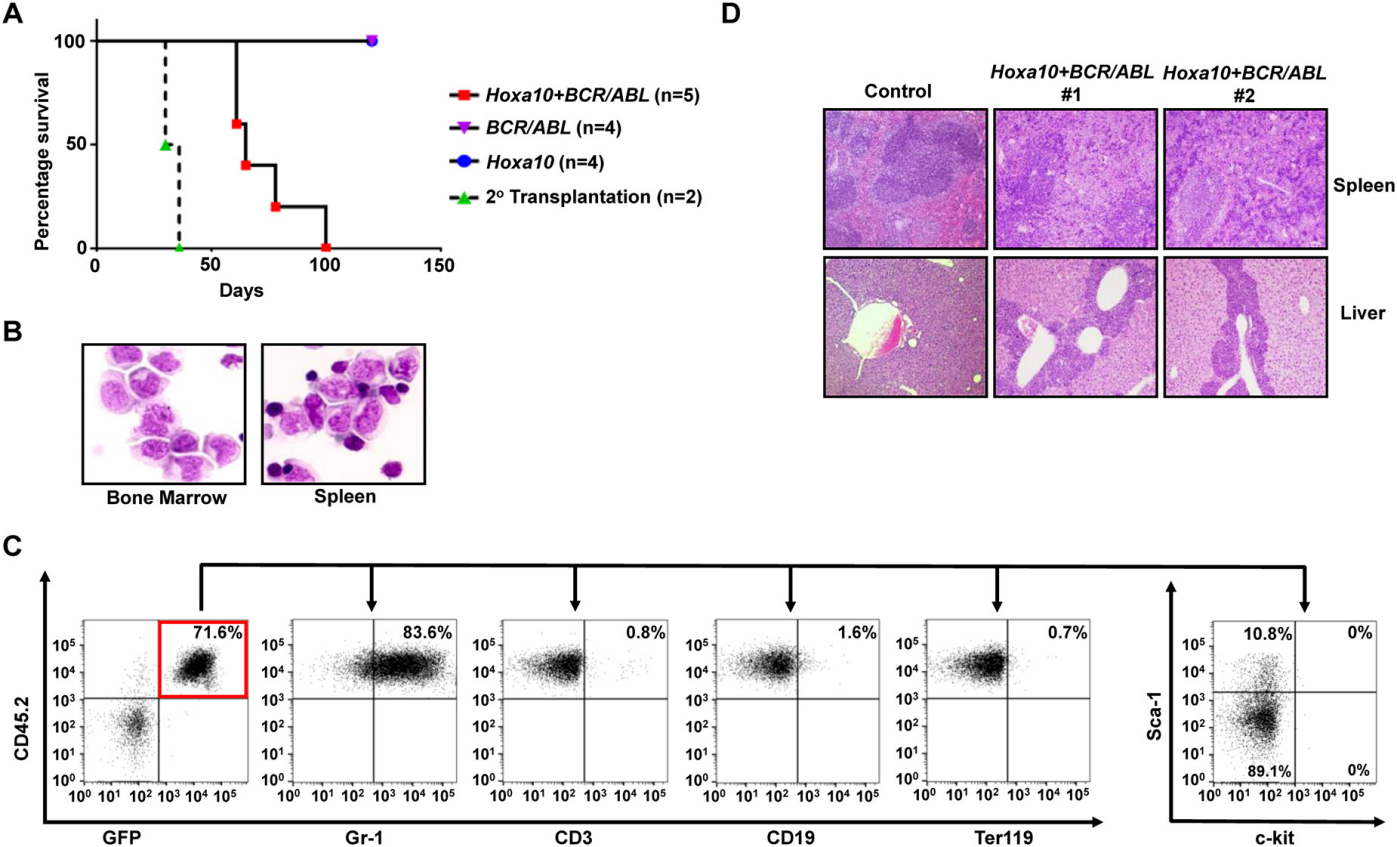
*Hoxa10* cooperates with *BCR/ABL* to induce development of CML myeloid blast crisis **(A)**. Survival curves of lethally-irradiated B6-Ly5.2 mice receiving GMPs transduced with *MSCV-Hoxa10-PGK-Puro* virus alone, *MSCV-BCR/ABL-IRES-GFP* virus alone, the combination of both viruses, or 1 × 10^6^ spleen cells from primary leukemic mice. **(B)**. Representative cytospin analysis of bone marrow and spleen cells from leukemic mice. **(C)**. Representative FACS analysis of GFP and CD45.2 double positive leukemia cells from the bone marrow of leukemic mice using the indicated antibodies. Numbers represent the percentages of gated events. **(D)**. H&E staining of spleen and liver tissue sections showing leukemic infiltration in two *Hoxa10+BCR/ABL* leukemic mice in comparison to a healthy control mouse.

Utilizing the same approach, we also examined the capability of *Hoxa10* to cooperate with *BCR/ABL* to transform normal GMPs *in vivo*. Interestingly, we found that all mice receiving GMPs co-transduced by *BCR/ABL* and *Hoxa10* viruses also developed myeloid leukemias, although with longer latencies than leukemias induced by *Hoxa9+BCR/ABL* (Figure 3A). Again, none of the recipient mice for GMPs singly infected with *Hoxa10* or *BCR/ABL* virus developed leukemia (Figure 3A). Similar to leukemias induced by *Hoxa9+BCR/ABL*, these leukemias also are characterized by expansion and infiltration of myeloid blasts into the bone marrow, spleen, and liver (Figure 3B and 3D). Myeloid blasts represent 25 ± 1.4% (Mean ± SD) of all nucleated cells in the bone marrow, suggesting development of myeloid blast crisis. Consistent with their myeloid origins, the leukemia cells are also mostly positive for Gr-1 expression and negative for the expression of CD3, CD19, and Ter119 (Figure 3C). Additionally, less than 1% and 6 to 10% of the leukemia cells expressed c-kit and Sca-1, respectively (Figure 3C). Consistent with their co-transduction by *BCR/ABL* and *Hoxa10* viruses, these leukemia cells expressed high levels of *BCR/ABL* and *Hoxa10* (Supplementary Figure 1). These leukemias also are transplantable, as secondary transplantation of the leukemia cells led to development of the same disease in recipient mice in 40 days (Figure 3A). All puromycin-resistant colonies formed by the leukemia cells also were GFP positive (data not shown), suggesting that both need to be expressed in the same cells to induce transformation. These data suggest that overexpression of *Hoxa10* is also able to cooperate with *BCR/ABL* to induce CML myeloid blast crisis.

### *Myb* expression is essential for *Hoxa9* and *Hoxa10*-induced self-renewal

**Figure 4:**
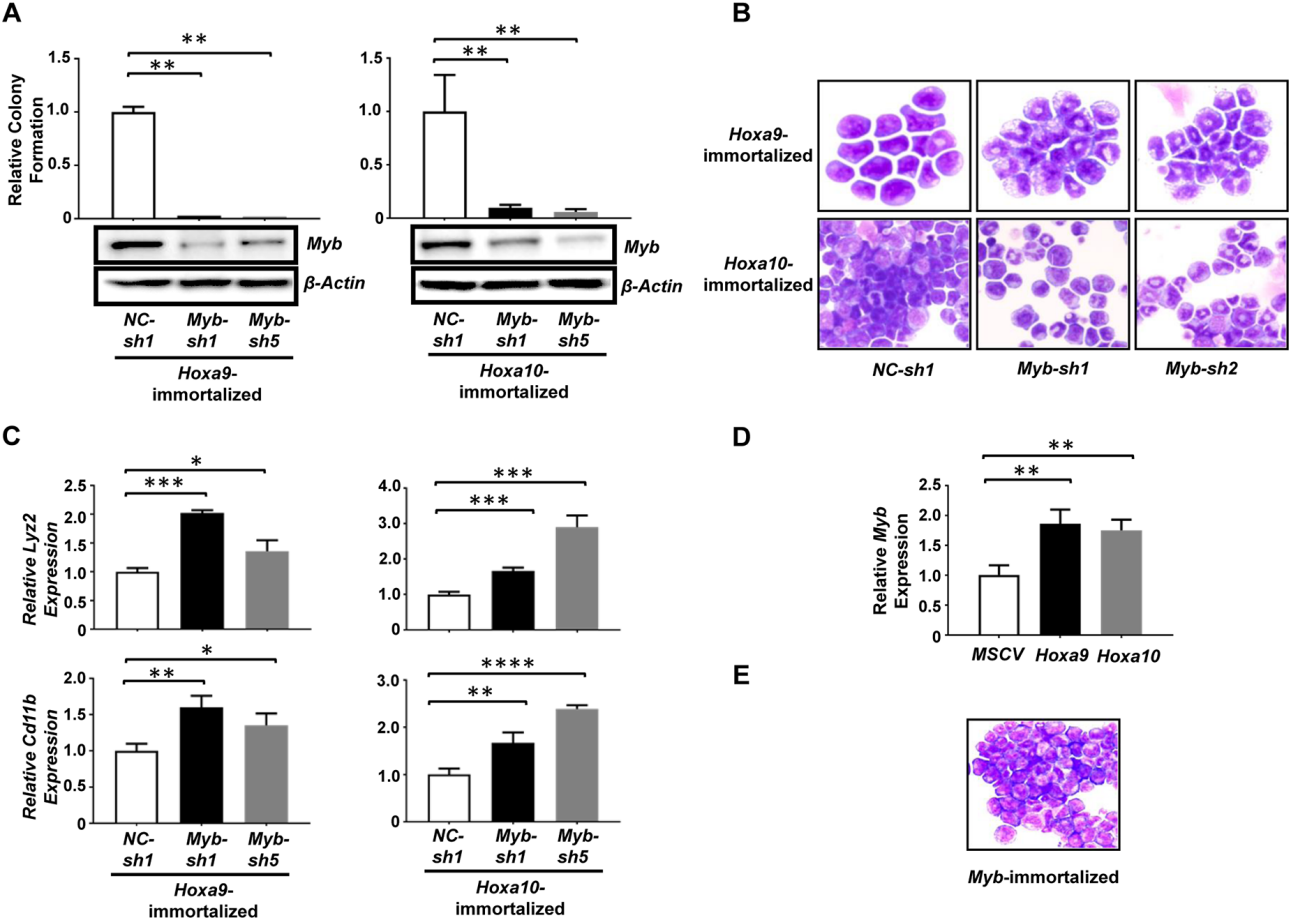
*Myb* is a critical mediator of *Hoxa9/Hoxa10*-induced self-renewal of myeloid progenitors **(A)**. Upper panels, colony-forming potential of myeloid progenitors immortalized by *Hoxa9* (left) and *Hoxa10* (right) plated 48hrs after infection with lentiviral shRNA targeting *Myb* (*Myb-sh1*, *Myb-sh5*) or control shRNA (*NC-sh1*). Lower panel, Western blotting analysis of Myb protein at 72hrs after infection in the same cells corresponding to the upper panel. **(B)**. Cytospin analyses of *Hoxa9* (top) and *Hoxa10*-immortalized cells (lower panels) 72hrs after infection with the indicated lentiviral shRNAs. **(C)**. Real-time PCR analysis of *Lyz2* and *Cd11b* mRNA levels in either *Hoxa9* or *Hoxa10*-immortalized myeloid progenitor cells 72hrs after infection with the indicated lentiviral shRNAs. Relative expression levels were calculated by normalizing to *β-Actin* mRNA levels in the same sample and also in cells infected by *NC-sh1*. **(D)**. Real-time PCR analysis of *Myb* mRNA levels in mouse 5-FU treated BM progenitor cells 72hrs after infection with the indicated retrovirus. Non-transduced cells were eliminated by selection with 2 ug/ml of puromycin added at 48 hrs after infection. Relative expression levels were calculated by normalizing to *β-Actin* mRNA levels in the same sample and also in cells infected by empty virus. The mean and SD of each relative expression level is shown. **(E)**. Representative cytospin analysis of *Myb*-immortalized cells. ^*^, *P* <0.05; ^**^, *P* < 0.01; ^***^, *P* < 0.001; ^****^, *P* < 0.0001 (two-tailed Student's *t* test).

The common capability of *Hoxa9* and *Hoxa10* to induce the self-renewal of myeloid progenitors suggests that they may activate the same target(s) critical for this process. Interestingly, we found that the expression of *Myb*, a helix-turn-helix transcription factor important for hematopoietic development and a critical target for *Hoxa9*-induced myeloid leukemia development [16], was up-regulated to similar levels in *Hoxa9+BCR/ABL* and *Hoxa10+BCR/ABL* leukemias (Supplementary Figure 2), suggesting that *Myb* could be a shared critical target of *Hoxa9* and *Hoxa10* in their stimulation of self-renewal. Therefore, we examined whether continuous expression of *Myb* is required for the self-renewal of myeloid progenitors immortalized by *Hoxa9* and *Hoxa10.* As expected, *Myb* knockdowns using two different *Myb*-specific lentiviral shRNAs dramatically reduced the colony-forming potential of *Hoxa9-*immortalized cells (Figure 4A). Similarly, infection with the same lentiviral shRNAs also markedly inhibited the colony formation by *Hoxa10*-immortalized cells (Figure 4A), suggesting that *Myb* expression is also critical for *Hoxa10*-induced self-renewal. Consistent with this idea, cytospin analysis revealed significant differentiation of both *Hoxa9* and *Hoxa10*-immortalized cells into neutrophils after *Myb* knockdown (Figure 4B), which was confirmed by significantly increased expression of myeloid differentiation markers including *Cd11b* and *Lyz2* (Figure 4C). To further test the possibility that *Hoxa10* could directly activate *Myb* transcription, we transduced 5-FU-treated mouse bone marrow progenitors with *Hoxa10*-expressing (*MSCV-Hoxa10-PGK-puro*), *Hoxa9*-expressing (*MSCV-Hoxa9-PGK-puro*) or empty virus (*MSCV-PGK-puro*), and compared the *Myb* expression levels in the transduced cells at 72hrs after infection following the elimination of non-transduced cells by selection with puromycin at 48hrs. Significant and comparable increases in *Myb* mRNA levels were observed in both *Hoxa9-* and *Hoxa10*-infected cells when compared to control cells infected with empty virus (Figure 4D), suggesting that *Myb* could be a direct transcriptional target of *Hoxa10*. In combination, these data suggest that *Myb* is a downstream target of both *Hoxa9* and *Hoxa10*, and also a critical mediator of their self-renewal promoting activity.

### *Myb* is capable of inducing the self-renewal of myeloid progenitors *in vitro* and *in vivo*

**Figure 5:**
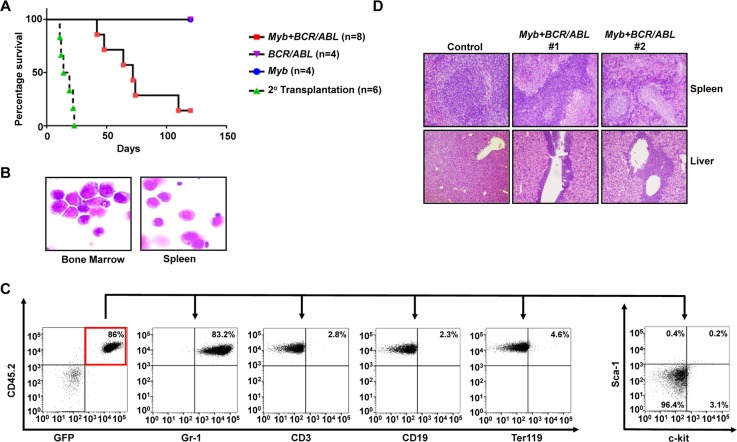
*Myb* is capable of cooperating with *BCR/ABL* to induce development of CML myeloid blast crisis **(A)**. Survival curves of lethally-irradiated C57BL6-*Ly5.2* mice receiving GMPs transduced with *MSCV-Myb-IRES-GFP* virus alone, *MSCV-BCR/ABL-IRES-GFP* virus alone, the combination of both viruses, or 1 × 10^6^ spleen cells from primary leukemic mice. **(B)**. Representative cytospin analysis of bone marrow and spleen cells from leukemic mice. **(C)**. Representative FACS analysis of GFP and CD45.2 double positive leukemia cells from the bone marrow of leukemic mice using the indicated antibodies. Numbers represent the percentages of gated events. **(D)**. H&E staining of spleen and liver tissue sections showing leukemic infiltration in two *Myb+BCR/ABL* leukemic mice in comparison to a healthy control mouse.

*Myb* has been indicated as an important self-renewal regulator for normal hematopoietic stem cells [17]. A role of *Myb* overexpression in promoting self-renewal was also suggested by the generation of an immortalized cell line from mouse fetal liver cells transduced by a mutant retrovirus expressing wild-type *Myb* [18]. To further understand the role of *Myb* activation in *Hoxa9-* and *Hoxa10*-induced myeloid progenitor self-renewal, we next tested if ectopic expression of *Myb* also could induce self-renewal of primary myeloid progenitors. Alternative splicing of *MYB* transcript results in the production of two isoforms: a predominantly expressed 75KDa form and a less expressed 89KDa form [19]. The 75KDa form also is predominantly expressed in *Hoxa9-* and *Hoxa10-*immortalized myeloid progenitors and *Hoxa9/Hoxa10*+*BCR/ABL* leukemia cells (data not shown). Therefore, we cloned the cDNA for the 75KDa *Myb* isoform into MSCV and tested the ability of the resulting virus (*MSCV-Myb-IRES-GFP*) to induce self-renewal of myeloid progenitors in culture under the same condition tested for *Hoxa9* and *Hoxa10* viruses. We found that expression of this *Myb* isoform is able to efficiently induce immortalization of myeloid progenitors (Figure 4E and Supplementary Figure 3), suggesting that it could confer unlimited self-renewal capability to myeloid progenitors. Cooperation between *Myb* and *Abl* activation in AML development has been suggested previously [20]. We next examined if *Myb* could cooperate with *BCR/ABL in vivo* to cause leukemic transformation of GMPs. Similar to our earlier studies, purified GMPs from C57BL/6 mice were co-transduced, with *BCR/ABL* and *Myb* virus, and subsequently transplanted into lethally irradiated B6-Ly5.2 mice. GMPs transduced by *BCR/ABL* or *Myb* virus alone were transplanted as controls. Interestingly, six out of eight mice receiving GMPs co-transduced with *BCR/ABL-* and *Myb*-expressing viruses developed myeloid leukemia in six months after transplantation while mice transplanted with GMP cells that were singly transduced with either *BCR/ABL* or *Myb* expressing retrovirus remained healthy during the same period (Figure 5A). Resembling mice with *Hoxa9+BCR/ABL* and *Hoxa10+BCR/ABL* leukemias, the bone marrow, spleen and liver of the moribund mice were infiltrated by immature leukemia blasts (Figure 5B and 5D), representing 31 ± 5.7% (Mean ± SD) of all nucleated cells in the bone marrow. These leukemia cells also displayed a similar lineage marker expression profile to that of *Hoxa9+BCR/ABL* and *Hoxa10+BCR/ABL* leukemia cells and were transplantable (Figure 5C and 5A). As expected from the co-transduction, these cells expressed significantly elevated levels of *Myb* and *BCR/ABL* mRNA (Supplementary Figure 4). Both *Myb* and *BCR/ABL* proviruses were detected in single methylcellulose colonies formed by the leukemia cells by PCR (data not shown), further suggesting that the transformation requires their expression within the same cell. In addition, these leukemia cells did not express detectable levels of Hoxa9 or Hoxa10 protein (Supplementary Figure 5), suggesting that their transformation is independent of *Hoxa9* or *Hoxa10* activation. Taken together, these results indicate that overexpression of *Myb* is also capable of inducing CML progression by inducing self-renewal of GMPs, although with a lower potency than *Hoxa9* and *Hoxa10*.

### Increased *Myb* expression is detected in CML blast crisis patients

**Figure 6:**
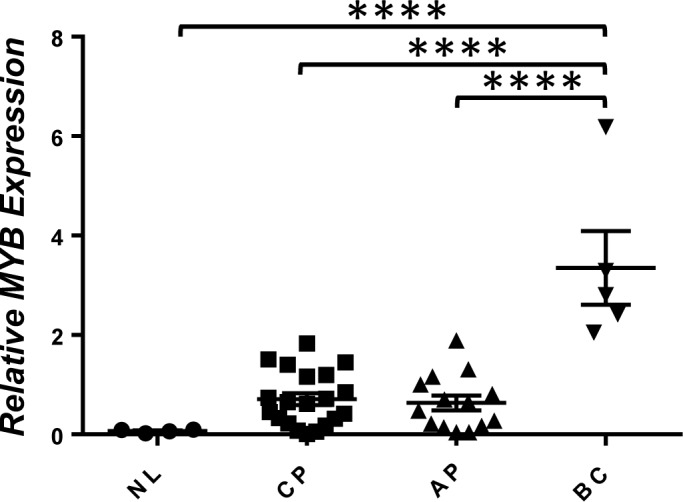
Increased expression of *MYB* in CML blast crisis patients Real-time RT-PCR analysis of *MYB* mRNA levels in total RNA isolated from whole bone marrow of healthy volunteers (NL) and CML chronic phase (CP), advanced phase (AP), and blast crisis phase (BC) patients. Relative expression levels were calculated by normalizing to *BCR* mRNA levels in the same sample. ^****^, *P* < 0.0001 (two-tailed Student's *t* test).

The ability of *Myb* to cooperate with *BCR/ABL* to induce transformation of GMPs into LICs *in vivo* suggests that its activation may play an important role in the progression of human CML. To test this hypothesis, we examined *MYB* mRNA levels in bone marrow aspirates of normal and CML patients at different stages of CML progression. Relatively low levels of *MYB* mRNA were observed in the BM of normal human volunteers, chronic phase CML patients, and accelerated phase patients. In contrast, significantly higher levels of *MYB* mRNA were detected in all myeloid blast crisis CML patients (Figure 6). This result suggests a possibility that *MYB* overexpression could be a major contributor to LIC self-renewal in blast crisis progression of CML patients.

## DISCUSSION

The molecular mechanisms underlying CML myeloid blast crisis development are poorly understood. It has been suggested that LICs in these leukemias are derived from GMPs with unlimited self-renewal capability [3]. *BCR/ABL* alone is not capable of stimulating the self-renewal of GMPs [1], suggesting that additional mutations must be acquired to confer self-renewal capability to chronic phase GMPs and to drive disease progression into myeloid blast crisis. We have shown previously that overexpression of *Setbp1* can cooperate with *BCR/ABL* to induce development of CML myeloid blast crisis by stimulating the self-renewal of GMPs [10]. To gain further insights into the mechanisms inducing GMP self-renewal in CML blast crisis, we tested whether overexpression of *Hoxa9* or *Hoxa10*, both critical targets of *Setbp1*, is sufficient to confer unlimited self-renewal capability to myeloid progenitors and to induce the development of CML blast crisis. Previous studies have shown that purified mouse GMPs transduced with retrovirus expressing either *Hoxa9* or *Hoxa10* could be serially plated multiple times in colony assays [14]; however, the full potential of their expression in inducing long-term self-renewal of myeloid progenitors *in vitro* and *in vivo* remained to be determined. Our results strongly suggest that overexpression of either homeobox gene alone is adequate to confer unlimited self-renewal capability to myeloid progenitors *in vitro* as transduction of these cells with even low titers of retroviral vectors expressing either gene efficiently induced their immortalization in culture. In addition, as normal mouse GMPs differentiate quickly and do not engraft in recipient mice after transplantation [21], the leukemic transformation of these cells induced by co-expression of *Hoxa9*/*Hoxa10* and *BCR/ABL* in our transplantation studies further suggests that both *Hoxa9* and *Hoxa10* activation also are able to confer unlimited self-renewal capability to chronic phase GMPs *in vivo*. Although both *HOXA9* and *HOXA10* were previously found to be overexpressed in CML blast crisis patients [8], their roles in CML progression have been unclear. Our *in vivo* results strongly identify *HOXA9* and *HOXA10* as potent drivers of human CML myeloid blast crisis development.

Different from GMP-derived leukemias induced by *Setbp1* and *BCR/ABL* [10], *Hoxa9+BCR/ABL* and *Hoxa10+BCR/ABL* leukemias are mostly monoclonal in origin (Supplementary Figure 6), suggesting that additional mutation(s) is likely required for the transformation in both cases. These mutations may be required for further stimulation of proliferation and/or inhibition of apoptosis of GMPs. Our results also suggest that *Hoxa9* is more potent than *Hoxa10* in inducing CML myeloid blast crisis, as the *Hoxa9+BCR/ABL* mice developed leukemia with a significantly shorter latency than *Hoxa10+BCR/ABL* mice. This could be due to less mutations required for GMPs to become transformed after transduction by *Hoxa9* and *BCR/ABL* viruses or the potential inhibitory effect of high levels of *Hoxa10* expression on cell proliferation described previously [22].

*Myb* is a master regulator of normal hematopoietic development [17, 23]. In addition, *Myb* is a significant contributor to leukemogenesis as it has been shown to be a critical target of multiple known oncogenes in the hematopoietic system, including *HOXA9* [16], *MLL* fusions [24], and recently *Setbp1* [25]. Our study additionally identifies *Myb* as a critical target of *Hoxa10* for its self-renewal stimulating activity, as *Myb* knockdown caused differentiation of *Hoxa10*-immortalized myeloid progenitors. It remains unclear how *Hoxa10* activates *Myb* transcription. Studies have suggested that Hoxa9 can directly activate *Myb* transcription through binding to regions of *Myb* promoter and first intron, and also a recently identified distal regulatory element [26, 27]. It is possible that the same sites could be responsible for mediating the activation by *Hoxa10*, as Hox proteins are known to recognize similar binding sites [28].

Previous studies have suggested a critical requirement for *MYB* in the maintenance of CML cells. Inhibition of *MYB* expression by anti-sense oligonucleotides previously has been shown to reduce the expansion of human CML blast crisis cells *in vitro* [29]. Genetic reduction in *Myb* levels also has been found to suppress *BCR/ABL*-induced CML development [30]. It was unclear, however, whether *MYB* plays any causal role in driving CML progression. Our bone marrow transplantation studies show for the first time that overexpression of *Myb* is capable of inducing the development of CML myeloid blast crisis *in vivo*, when expressed together with *BCR/ABL* in GMPs. Our *in vitro* immortalization studies further indicate that this capability of *Myb* is likely due to its ability to induce self-renewal of myeloid progenitors, which is consistent with its potential function in maintaining the self-renewal of hematopoietic stem cells during normal hematopoietic development [17]. The incomplete penetrance of the leukemia development induced by *Myb* and *BCR/ABL* and the monoclonal origins of the resulting leukemias (Supplementary Figure 6) also suggest that additional mutations, potentially with effects of increasing proliferation and/or inhibiting apoptosis, are required for the transformation of GMPs. Significantly higher levels of *MYB* expression also were detected in all CML myeloid blast crisis patients than chronic and accelerated phase cases examined in our study, further suggesting a possibility that abnormal activation of *MYB* expression could be a major “driver” for CML progression into myeloid blast crisis by conferring self-renewal capability to GMPs. Therefore, our study also suggest that strategies to block MYB activity may prove effective for inhibiting LIC self-renewal in CML blast crisis, for which effective treatments are lacking.

## MATERIALS AND METHODS

### Mice

7-12 week-old C57BL/6 and B6-Ly5.2 mice were purchased from Charles River, Frederick, MD. These mice were maintained in the animal facility of Laboratory of Animal Medicine at Uniformed Services University of the Health Sciences (USUHS). All mouse experiments were carried out according to protocols approved by the USUHS Institutional Animal Care and Use Committee.

### Patient samples

Primary human cells were collected after signing the informed consent, according to the protocols approved by the Institutional Review Board of City of Hope (COH) or the University of Alabama at Birmingham (UAB), in accordance with assurances filed with the Department of Health and Human Services, and met all requirements of the Declaration of Helsinki.

### Retrovirus generation

*MSCV-BCR/ABL-IRES-GFP* and *MSCV-Hoxa9-PGK-Neo* viruses have been described previously [11, 31]. For the generation of *MSCV-Hoxa10-PGK-Puro* and *MSCV-Myb-IRES-GFP* retroviral constructs, cDNAs for Hoxa10 and the 75 KDa form of Myb were amplified by RT-PCR, confirmed by sequencing, and cloned into *MSCV-PGK-Puro* and *MSCV-IRES-GFP* vectors, respectively. The primers utilized were: *Hoxa10* S, 5’-CGC GGG ATC Ccc cac aac aat gtc atg ctc -3’; *Hoxa10* AS, 5’-CGC GGA ATT CGC GAA AAG ACG TTG TCT GGA AG -3’; *Myb* S, 5’-CGC GCT CGA GCC TCG CCA TGG CCC GGA GAC -3’; *Myb* AS, 5’-CGC GGC GGC CGC GGA AAT GTC TCA CAT GAC CA-3’. To generate retroviral construct *MSCV-Hoxa9-PGK-Puro*, *Hoxa9* cDNA was excised from *MSCV-Hoxa9-PGK-Neo* by *Xho*I/*EcoR*I double digestion and subsequently ligated to the same restriction sites in *MSCV-PGK-Puro* vector. Viruses were produced by the transfection of the *Hoxa9, Hoxa10, BCR/ABL*, *Myb* retroviral plasmids into Plat-E cells using Fugene 6 (Promega, Madison, WI). Viral titers were determined by infecting NIH3T3 cells.

### Immortalization of myeloid progenitors

Immortalization of myeloid progenitors was carried out as described [10]. Briefly, bone marrow cells harvested from C57BL/6 mice were cultured first in Stemspan medium (Stemcell Technologies Inc., Cambridge, MA) containing mouse SCF, TPO, IGF-2, and human FGF-1 for six days to expand hematopoietic stem cells, and then in IMDM plus 20% heat-inactivated horse serum with mouse SCF and IL-3 (Biolegend, San Diego, CA) for four days to induce production of myeloid progenitors. 5 × 10^5^ resulting cells were then infected with retrovirus (1 × 10^5^ cfu) on plates coated with Retronectin (Takara Bio USA, Mountain View, CA) for 48 hours. Infected cells were then continuously passaged at 1:10 ratio every three days for at least four weeks to assess immortalization of myeloid progenitors.

### Retroviral transduction and transplantation of GMPs

GMPs purified from C57BL/6 mice (8-12 weeks-old females) were transduced and subsequently transplanted as described previously. Briefly, freshly sorted GMPs were infected immediately with retrovirus at 4:1 ratio of viral titer to cell number on Retronectin-coated plates in the presence of mouse SCF (100ng/ml) and IL-11(10ng/ml). After 24 hours, a second infection was carried out under the same condition to increase transduction efficiency. At 48 hours after the first transduction, transduced cells were transplanted into B6-Ly5.2 recipient mice (7-12 weeks-old females) at 1.5 × 10^5^ cells per recipient along with supporting bone marrow cells (7.5 × 10^5^ cells/recipient) via tail vein injection. Before injection, recipient mice were irradiated twice at a total dose of 1100 rads from a ^137^Cs source. For secondary transplantation, 1 × 10^6^ spleen cells from primary recipients with leukemia were injected into lethally irradiated secondary recipients along with 7.5 × 10^5^ supporting bone marrow cells.

### Flow cytometry

Purification of mouse GMPs (IL-7RαˉSca-1ˉ c-Kit+FcγR-II/III^Hi^CD34+) and flow cytometry analyses of immortalized myeloid progenitors and leukemic bone marrow and spleen cells were performed as described previously [10].

### Lentiviral production, infection, and analysis

*Myb* shRNAs (*Myb-sh1* and *-sh5*) were described previously [25] and cloned into the pLKO.1-blast lentiviral vector [32]. pLKO.1-blast-SCRAMBLE [32] was used as the negative control (*NC-sh1*). Both pLKO.1-blast and pLKO.1-blast-SCRAMBLE were gifts from Dr. Keith Mostov (Addgene plasmid #26655 and #26701). To generate infectious lentivirus, the constructs were co-transfected using Fugene 6 (Promega, Madison, WI) into 293T cells along with packaging plasmid Δ8.9 and a plasmid expressing VSV-G, and virus was harvested at 72 hours after transfection. Viral titers were calculated by infecting NIH-3T3 cells with serial dilutions of viral stocks and isolating blasticidin resistant colonies. Lentiviral infections were performed by spinoculation in which a mixture of lentivirus and target cells at 4:1 ratio in 48-well or 24-well plates were centrifuged at 2000 x g for 90 minutes at 37°C. Blasticidin (12 μg/ml) was added to the infected cells 24 hours after infection. Colony formation assays were performed at 48 hours after infection using 1 x10^4^ blasticidin-resistant cells on IMDM methylcellulose medium supplemented with 15% fetal bovine serum, mouse SCF (50ng/ml), IL-3 (6ng/ml), and blasticidin (12 μg/ml). Colony numbers were counted after seven days.

### Western blotting analysis

Western blotting analyses were carried out as previously described [10]. Immunoblotting was carried outusing anti-Hoxa9 (07-178, Merck Millipore, Billerica, MA), anti-Hoxa10 (SC-17159, Santa Cruz Biotechnology, Santa Cruz, CA), anti-Myb (05-175 Millipore), β-Actin (MAB1501R, Millipore) primary antibodies, and goat anti-rabbit IgG-HRP (sc-2004, Santa Cruz Biotechnology) and rabbit anti-mouse IgG-HRP (a-9044, Sigma Aldrich, St. Louis, MO) secondary antibodies. Protein bands were visualized by incubation with SuperSignal West chemi-luminescent substrate (Pierce, Thermo Fisher Scientific, Waltham, MA).

### Real-time RT-PCR

For mouse cells or tissues, total RNA was extracted using RNAeasy Plus mini kit (Qiagen, Germantown, MD). Oligo-dT-primed cDNA samples were prepared from total RNA using Superscript III (Life Technologies, Carlsbad, CA), and real-time PCR analysis was performed in triplicates using SYBR green detection reagents (Life Technologies) according to the manufacturer's instructions in 20-μL final volume on a 7500 real time PCR system (Applied Biosystems, Foster City, CA). Relative changes in expression of *BCR/ABL*, *Hoxa9, Hoxa10, and Myb* were calculated according to the ΔΔCt method. The cycling conditions were 50°C for 2 minutes, followed by 95°C for 2 minutes, and then 40 cycles of 95°C for 15 seconds and 60°C for 1 minute. The following gene-specific primer sequences were used: *BCR/ABL* S, 5’-TCC GCT GAC CAT CAA YAA GGA-3’; *BCR/ABL* AS, 5’- CAC TCA GAC CCT GAG GCT CAA-3’; Hoxa9 S, 5’-TGT CTC CTC TCC CCC AAA CC-3’; Hoxa9 AS, 5’-GAG ATG AGG CCT GGG ATTTAG A-3’; Hoxa10 S, 5’-CCA CAG GCC ACT TCG TGT T-3’; Hoxa10 AS, 5’-TCG TAG AGG CAG TAG GAG CTC TCT-3’; Myb S, 5’-CCA TGA AAG CTC GGG CTT AG-3’; Myb AS, 5’-CTC GAC ATG GTG TCA GTT GTG-3’; Rpl4 S, 5’-ATG ATG AAC ACC GAC CTT AGC A-3’; Rpl4 AS, 5’-CGG AGG GCT CTT TGG ATT TC-3’.

For human bone marrow cells, total RNA was extracted with TRizol (Life Technologies) according to manufacturer's instruction. First-strand cDNA was synthesized using the Superscript III first strand kit (Life Technologies). All reactions were performed in a total volume of 20 μl. Quantitative detection of *MYB* and *BCR* transcripts was performed by q-PCR analysis using a TaqMan universal PCR master mix kit and the ABI Prism 7900 sequence detector (Applied Biosystems). TaqMan® Gene Expression Assays probes were purchased from Applied Biosystems. The housekeeping gene *BCR* was measured as an internal control. The levels of *MYB* were calculated based on standard curves and expressed as relative ratio to *BCR*.

## SUPPLEMENTARY MATERIALS FIGURES


