# Supplementary material for: Hoxa9 and Hoxa10 induce CML myeloid blast crisis development through activation of Myb expression
Source: Oncotarget. 2017 Oct 24;8(58):98853–64. doi: 10.18632/oncotarget.22008 (PMC5716772; doi:10.18632/oncotarget.22008)
Supplement: Supplementary file 1 [file oncotarget-08-98853-s001.pdf]

Hoxa9 and *Hoxa10* induce CML myeloid blast crisis development through activation of *Myb* expression

SUPPLEMENTARY MATERIALS

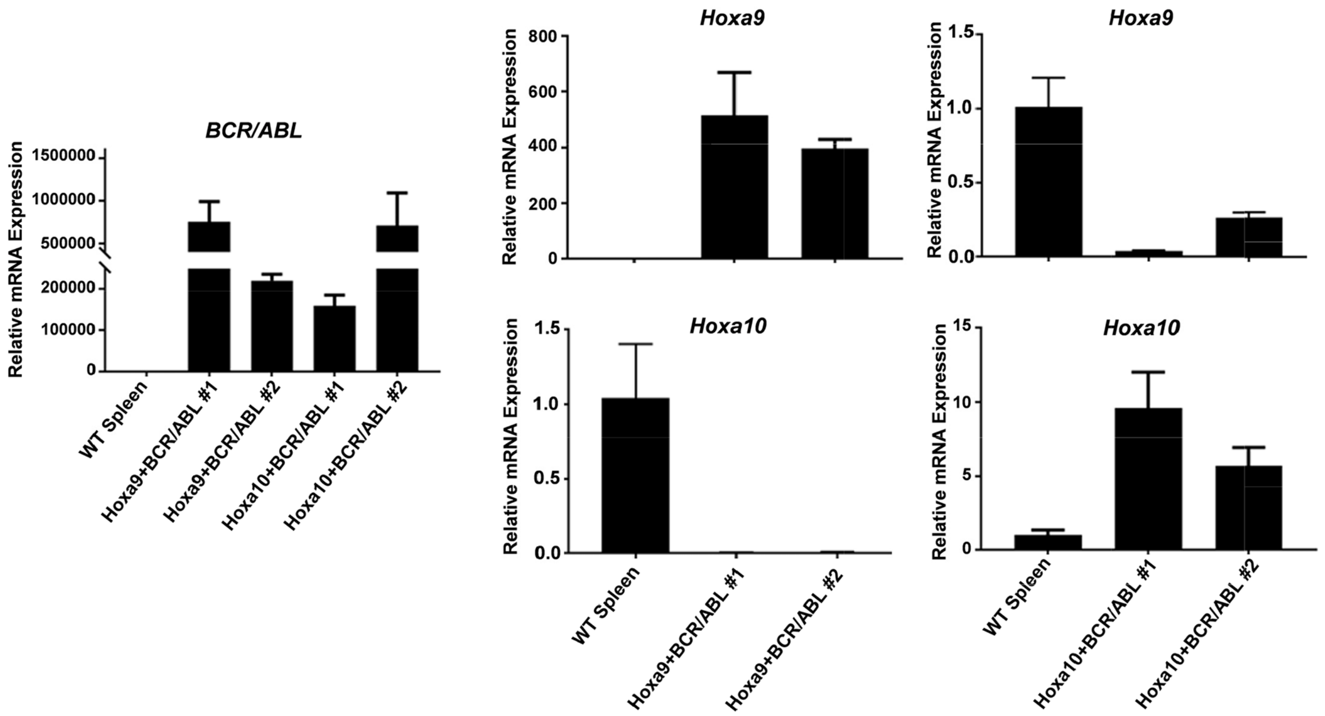

Supplementary Figure 1: Expression of *BCR/ABL*, *Hoxa9*, and *Hoxa10* mRNAs in *Hoxa9+BCR/ABL* and *Hoxa10+BCR/ABL* leukemias. Real time RT-PCR analyses of *BCR/ABL*, *Hoxa9* and *Hoxa10* mRNA levels in indicated Hoxa9+BCR/ABL and Hoxa10+BCR/ABL leukemic spleens in comparison to a wild-type spleen. Relative expression levels were calculated by normalizing to *β-Actin* mRNA levels in the same sample and also in wild-type spleen. The mean and SD of each relative expression level is shown. No *BCR/ABL* mRNA was detected in wild-type spleen and a Ct value of 40 was assigned to the sample for the calculation of relative *BCR/ABL* expression levels in the leukemia samples.

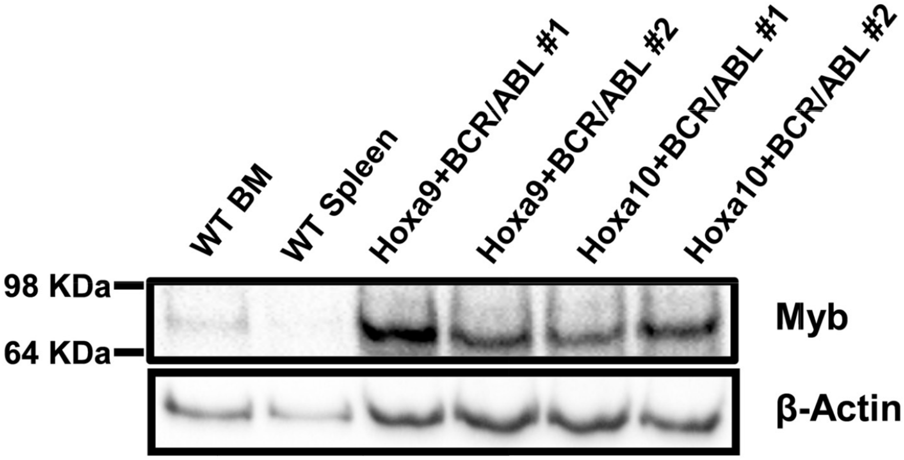

Supplementary Figure 2: Increased levels of Myb protein in *Hoxa10+BCR/ABL* leukemias. Western blotting analyses of protein extracts prepared from spleens of indicated Hoxa10+BCR/ABL leukemic mice (n=2), Hoxa9+BCR/ABL leukemic mice (n=2), and wild-type spleen and bone marrow cells using the indicated specific antibodies.

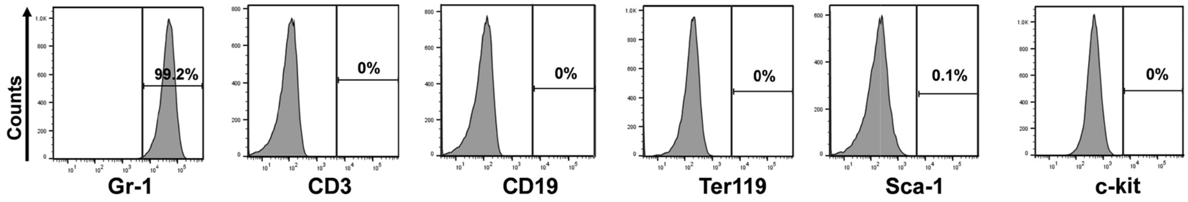

Supplementary Figure 3: Surface marker expression by *Myb*-immortalized cells. Representative FACS analysis of indicated marker expression of cells immortalized by transduction with retroviruses expressing *Myb* short isoform after passaging in liquid media containing SCF and IL-3 for two months. Numbers represent the percentages of gated events.

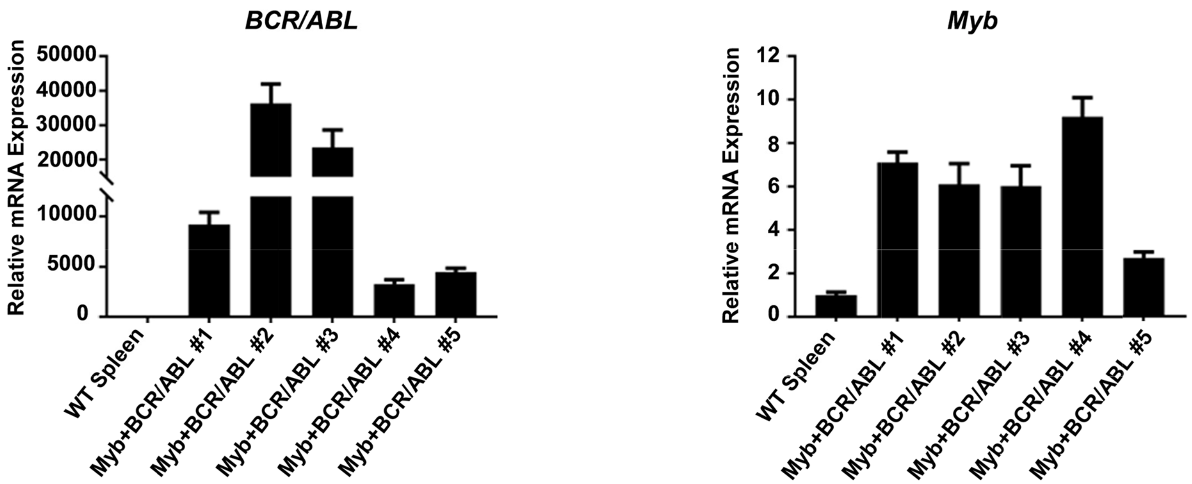

Supplementary Figure 4: Expression of *BCR/ABL* and *Myb* mRNAs in *Myb+BCR/ABL* leukemias. Real time RT-PCR analyses of *BCR/ABL* (Left panel) and *Myb* (Right panel) mRNA levels in indicated *Myb+BCR/ABL* leukemic spleens in comparison to a wild-type spleen. Relative expression levels were calculated by normalizing to *β-Actin* mRNA levels in the same sample and also in wild-type spleen. The mean and SD of each relative expression level is shown. No *BCR/ABL* mRNA was detected in wild-type spleen and a Ct value of 40 was assigned to the sample for the calculation of relative *BCR/ABL* expression levels in the leukemia samples.

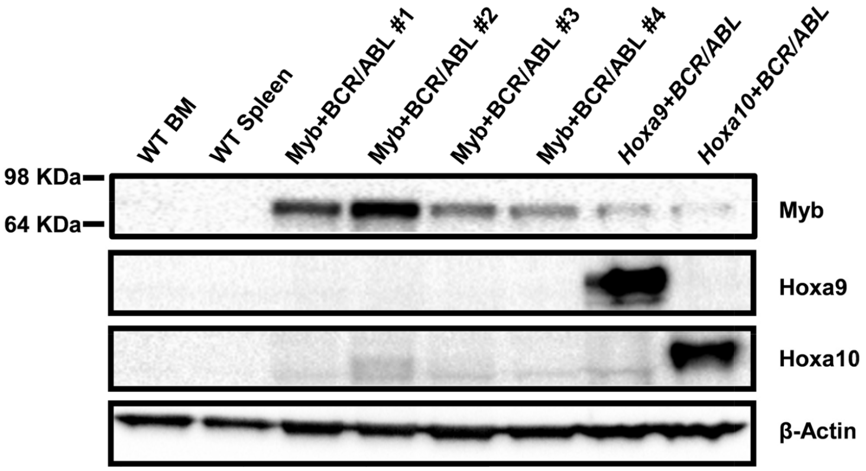

Supplementary Figure 5: *Myb+BCR/ABL* leukemias do not express significant levels of Hoxa9 and Hoxa10 proteins. Western blotting analyses of protein extracts prepared from leukemic spleens of indicated *Myb+BCR/ABL* leukemias (n=4), *Hoxa9+BCR/ABL* leukemia (n=1), and *Hoxa10+BCR/ABL* leukemia (n=1), and wild-type spleen and bone marrow cells using the indicated specific antibodies.

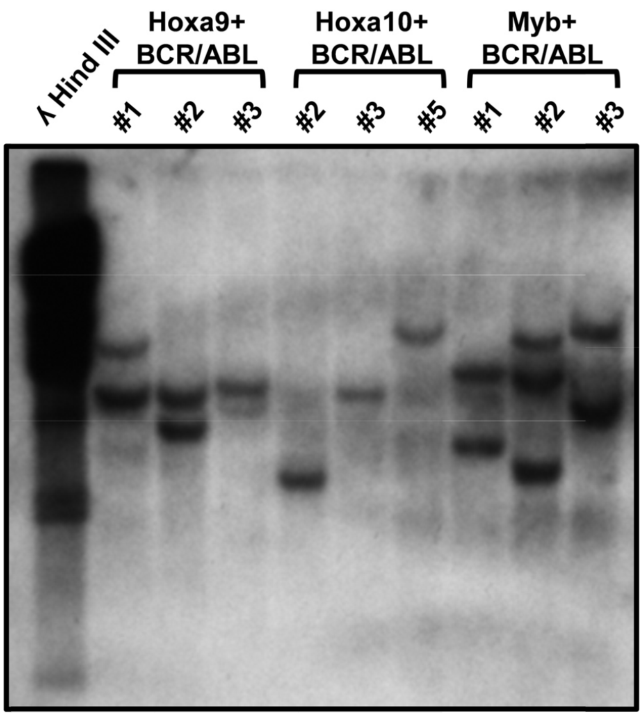

Supplementary Figure 6: *Hoxa9+BCR/ABL*, *Hoxa10+BCR/ABL*, and *Myb+BCR/ABL* leukemias are mostly monoclonal. Southern blotting analysis of genomic DNA from the indicated leukemic spleens using a *GFP*-specific probe for the detection of *BCR/ABL* and *Myb* integrations. Samples were digested by *Eco*RI, and each band represents a separate integration. Note that the leukemia samples have either a single integration band or integration bands of similar intensity, suggesting that they contain one or one dominant leukemic clone.
